# Brief communication: Effect of mobile health intervention on medication time adherence among people living with HIV/AIDS receiving care at selected hospitals in Owerri, Imo State Nigeria

**DOI:** 10.1186/s12981-024-00653-0

**Published:** 2024-10-24

**Authors:** Chinelo Judith Ezelote, Eunice Anyalewechi Nwoke, Sally Nkechinyere Ibe, Blessed Okwuchi Nworuh, Gregory Ndubeze Iwuoha, Chimezie Christain Iwuala, Obinna Godwin Udujih, Joy Nkechi Osuoji, Alain Simon Inah, Alexis Ebikonbowei Okaba, Eleanor Asuzu

**Affiliations:** 1 Public Health Department, Federal University of Technology Owerri, Owerri, Imo State Nigeria; 2 Public Health Department, University of Calabar, Calabar, Cross River State Nigeria; 3 Department of Biological Sciences, University of Africa, Toru-Orua, Bayelsa State Nigeria

**Keywords:** HIV/AIDS, PLWHA, Medication-time adherence, M-Health, ART

## Abstract

**Aim:**

This study aimed to assess the impact of Mobile health (M-health) on medication time adherence among people living with HIV/AIDS (PLWHA).

**Methods:**

The study included all PLWHA who were receiving care at the Federal University Teaching Hospital Owerri (FUTH) and Imo State Specialist Hospital (ISSH) Umugumma during the study duration. The test group (FUTH) received a 2-way text message sent three times a week and a once-a-week phone call, while the control group (ISSH) received only the standard care.

**Findings:**

The result shows that the adherence was higher among PLWHA in the test group compared to those in the control group (*P* = 0.000, χ2 = 168.62, 95% confidence interval (CI): 7.22 to 16.19).

**Conclusion:**

M-health intervention significantly improved the medication time adherence among the participants in the test group compared to those in the control group.

**Supplementary Information:**

The online version contains supplementary material available at 10.1186/s12981-024-00653-0.

## Background

Mobile health (also written as M-health or mhealth) is an abbreviation for mobile health, a term used for the practice of medicine and public health supported by mobile devices [1]. Recent studies have focused on evaluating the various functions of smartphones. These studies have included an analysis of using built-in digital diaries for symptom research [2], utilizing SMS text messages for behavior change management [3], sexual health education [4], and enhancing patients’ adherence to antiretroviral treatment for PLWHA [5]. HIV treatment involves taking medicine known as antiretroviral therapy or ART as prescribed by a health care provider [6]. There is consistent evidence that very early ART may benefit the individual infected with HIV by leading to more rapid and robust immunologic recovery, lower inflammation and reduced viral reservoir size compared to a later start [7-10]. According to WHO guidelines, a high level of adherence (> 95%) is required for ART to be effective [11]. Medication adherence was also defined as taking all medications at the appropriate time with the appropriate dosage as prescribed by the physician [12]. In a meta-analysis of randomized controlled trials addressing the impact of M-health on adherence to ART among PLWHA, the researchers reached the conclusion that further investigation is necessary to ascertain the potential of M-health in enhancing ART adherence in PLWHA [13]. They further emphasized the need for additional efforts to effectively integrate M-health into the clinical administration of ART for individuals with HIV [14]. A systematic review and meta-analysis of randomized controlled studies conducted among PLWHA showed telehealth interventions were found to increase the adherence to treatment, reduce depressive symptoms, and improved perceived quality of life [14]. The researchers recommended further research to evaluate telehealth interventions among other customizable variables that affect intervention effectiveness. The study conducted on ART adherence in South Florida among 94 treatment-naïve patients showed that a one-way daily text messaging intervention did not improve ART adherence [15]. The researchers suggested conducting larger controlled studies to determine the potential benefit of this technology-based intervention on the impact of ART adherence for PLWHA in lower-resource settings. The findings of a qualitative study undertaken to investigate the perceptions and experiences of utilizing mobile technology to enhance medication adherence among elderly individuals with coronary heart disease indicated that text messaging and mobile phone applications are perceived as beneficial tools for promoting medication adherence [16]. It was recommended that robust clinical trials be conducted to evaluate the efficacy of mobile health technology in bolstering medication adherence among populations necessitating stringent medication adherence. This study aims to assess the effect of mobile health intervention on the medication-time adherence among people living with HIV/AIDS in selected hospitals in Imo State.

## Methods

A quasi-experimental study was undertaken on all the PLWHA who were receiving care at the Federal University Teaching Hospital (FUTH) in Owerri and Imo State Specialist Hospital in Umuguma (ISSH), Imo State, during the period from December 2023 to May 2024. At that time, the hospital management and the program officer in charge of the Heart to Heart Centre granted the researchers full access to PLWHA who were receiving care at their facilities. Selection of these hospitals was predicated on their established status, substantial population of HIV-infected patients undergoing ART, and their status as two of the largest healthcare facilities in Imo State grappling with a high incidence of HIV cases.

### Adherence measurement

Time-critical scheduled medications are those where early or delayed administration of maintenance doses of greater than 30 min before or after the scheduled dose may cause harm or result in substantial sub-optimal therapy or pharmacological effect, while non-time-critical scheduled medications are those where early or delayed administration within a specified range of either 1–2 h should not cause harm or result in substantial sub-optimal therapy or pharmacological effect [17-19] Medications prescribed for daily, weekly or monthly administration may be within 2 h before or after the scheduled dosing time, for a total window that does not exceed 4 h [19-20]. At the beginning of this study, participants selected a two-hour window each day for taking their ART medication, based on optimal intake times determined during their baseline assessments. After two months of intervention, this window was adjusted to one hour. The participants synchronized their medication intake with their daily routines, ensuring that it consistently occurred at the same time each day. Specifically, 97 participants chose to take their medication immediately upon waking, before getting out of bed. 53 opted to take it while brushing their teeth in the bathroom, 22 chose to take it while helping their kids with homework, and 51 preferred to take it while watching their favorite evening news or program. Participants took extra measures to ensure they didn’t miss their medication time, setting alarms on their clock, watch, or phone. Those who adhered to their medication schedule 95% of the time or more were considered to have good adherence, while those with lower rates were classified as non-adherents. The participants’ viral loads were measured at the beginning and end of the study.

### Sample size determination and sampling techniques

This study included all PLWHA who were receiving care at FUTH Owerri and ISSH Umuguimma who were non-adherent to their medication-time intake one year preceding this study. Out of the 1774 PLWHA at FUTH Owerri and 3650 PLWHA at ISSH Umugumma receiving care at the hospitals during this study, 223 and 411 were those non-adherent to their medication-time adherence respectively. No sampling was conducted as the entire population of non-adherents was included. However, one of the two facilities was chosen as the control using a simple random sampling method (by flipping a coin for heads or tails). The results showed that FUTH Owerri was the test group. Mobile health intervention was carried out in the test groups, while the control groups only received standard care. The hospitals were divided into test and control groups to conduct the m-health intervention in the test group.

### Data collection procedure

Adherence was assessed using a well-validated and structured questionnaire called the AIDS Clinical Trials Group (ACTG), adapted from a study by Reynolds et al. [21]. Participants reported their adherence by filling out the questionnaire, marking their calendars, and undergoing intermediate assessments once a week using a checklist. They were provided with a calendar to mark the days they adhered to their medication schedule. This information was calculated at the end of a four-month intervention period. Timely self-reported adherence was defined as a participant reporting that they had taken their ART medication within a 2-hour window. The questionnaire was administered to them at baseline and at the completion of the study. The data was gathered using a combination of face-to-face administration of questionnaire and online Google Form surveys. The research assistants, four health workers from the heart-to-heart centers, were university graduates between the ages of 20 and 30. They were trained by the principal researcher twice a week for two weeks and assisted in administering the questionnaire. If participants had any difficulty understanding the questions, the research assistants provided help. Any unanswered questions were referred to the principal researcher. The study lasted for six months, with four months dedicated to the intervention and an additional two months for participant follow-up.

### Study intervention

The intervention entailed the test group receiving M-health messages via SMS, phone calls, and WhatsApp voice notes. The participants received customized text messages three times a week to prompt them to take their ART medication as scheduled. The content of the messages was created by the researchers. The principal researcher personally contacted the participants once a week via phone calls to monitor their progress. Participants were sent medication-time adherence reminders for six days, followed by an assessment of their adherence level for the past six days on the seventh day using the checklist. This assessment was carried out every seventh day of the week to minimize recall bias. SMS reminders continued after the seventh day’s assessment. The overall assessment was conducted using the ACTG questionnaire and the personal calendar marked by the participants after the four-month intervention and two-month follow-up. At the end of the study, the participants’ overall percentage adherence levels were evaluated. A two-month follow-up without any intervention was conducted in all groups to assess participants’ medication time adherence post-intervention. Throughout the intervention, the participants were encouraged to respond to the researcher’s inquiries in an objective manner. They were instructed to direct any questions or feedback to the researcher or trained research assistants. Response times of 24 hrs on weekdays and 48 hrs on weekends were anticipated. For those who were unable to read or write, communication primarily took place through WhatsApp voice notes, using Nigeria’s ‘pidgin English’. The participants were assured of confidentiality and anonymity. The researcher utilized positive reinforcement, a crucial element of behavior change theory, to promote adherent behavior. This involved verbal praise, reward systems, added privileges, and other forms of positive feedback. Each communication concluded with a reminder for participants to take their medication regularly as scheduled to prevent relapse.

### Data processing and analysis

Statistical analyses were performed in SPSS version 29. T-test and Chi-square tests were used to establish relationship between the continuous and categorical variables respectively. Regression analysis was conducted to determine association between mobile health intervention and medication time adherence to antiretroviral medication between the test and control group. All association and statistical significance were measured using an odds ratio at a 95% confidence interval with a p-value of less than 0.05.

### Ethical considerations and informed consent

The study was conducted according to the guidelines of the Declaration of Helsinki and approved by the Health Research Ethics Committee of Federal Medical Centre (FMC), Owerri Imo State, Nigeria under the Chairmanship of Dr. I.I. Ike (FMC/OW/HREC/ Vol.11/048). All participants were informed about the purpose of the study and written informed consent was obtained before enrolment.

## Results

### Socio-Economic Information of the participants

In Table 1, the statistical analysis of the participants’ socio-economic data was presented using T-test and Chi-square methods. The analysis revealed that age, income, and duration on ART were not statistically significant, with p-values of 0.170, 0.510, and 0.318, and CI of -3.355 to 0.594, -8229.6 to 4096.4, and − 4.3 to 13.2, respectively. However, the viral load was found to be statistically significant, with a p-value of < 0.0001 and a CI of -1440.7 to -1076. Furthermore, based on the Chi-square analysis, marital status was found to be statistically significant with a p-value of < 0.0001, while gender was not statistically significant, with a p-value of 0.384.

**Table 1:** Socio-economic information of the participants at federal university teaching hospital Owerri and Imo state specialist hospital Umuguma

	FUTH		ISSH					95% Con. Int	
	Mean ± Std. Deviation		Mean ± Std. Deviation	Mean difference		P		lower	Upper
**Age**	37.4 ± 12.5		38.7 ± 11.5	-1.4		0.170		-3.355	0.594
**Income**	63144.8 ± 34741.7		65211.6 ± 37715.2	-2066.6		0.510		-8229.6	4096.4
**Duration on ART(months)**	86.2 ± 57.1		81.7 ± 49.8	4.4		0.318		-4.3	13.2
**Viral load**	325.6 ± 289.0		1584.4 ± 1335.7	-1258.7		< 0.0001		-1440.7	-1076.
**Chisquare analysis**									
**Gender**		**FUTH**	**ISSH**		**Chi square**		**p-value**		
Female		146 (65.5%)	283 (68.9%)						
Total		223	411		0.757		0.384		
**Marital Status**									
Married		72 (32.3%)	140 (34.1%)						
Single		83 (37.2%)	128 (31.1%)						
Cohabiting		9 (4.0%)	21 (5.1%)						
Widowed		10 (4.5%)	68 (16.5%)						
Separated		30 (13.5%)	44 (10.7%)						
Divorced		19 (8.5%)	10 (2.4%)						
Total		223	411		31.83		< 0.0001		

### Regression analysis of level of maintaining prescribed schedule of drug intake by the participants

Table 2 below shows that the adherence to maintaining a prescribed schedule of drug intake by the participants was higher among PLWHA at FUTH Owerri (test group) compared to those of the ISSS (control group). The p-value and CI were 0.000, and 7.22 to 16.19, respectively.

**Table 2:** Regression analysis of level of maintaining prescribed schedule of drug intake by the participants at federal university teaching hospital Owerri and Imo state specialist hospital Umuguma

Group	Adhered	Non-adhered	χ2	*p*	OR	Lower (95%CI)	Upper (95%CI)
Test (FUTH)	171(76.7%)	52(23.3%)					
Control(ISSH)	96 (23.4%)	315(76.6%)					
Total	267	367	168.62	0.000	10.79	7.22	16.19

## Discussion

At the end of this study, it was observed that participants in the test group showed a significant increase in medication adherence compared to those in the control group. Specifically, 76.7% of the test group maintained adherence, whereas only 23.4% of the control group did so. Statistical analysis revealed that the likelihood of non-adherence was nearly 11 times higher in the control group than in the test group. The intervention resulted in improved promptness in following the prescribed drug intake schedule among the participants. The viral loads of the participants were assessed after the intervention. The results of the viral load tested presented in Table 1 revealed a significant difference between the test and control groups, with the viral load of the participants in the test group decreasing significantly after the intervention compared to those in the control group. These findings align with those of other related studies, such as a study conducted among PLWHA at risk for disengaging with care at using M-health intervention at Miriam Hospital Immunology Center USA [22]; another study among PLWHA at risk for ART non-adherence using a novel M-health intervention named Rango [23]; research on the efficacy of a mobile phone–based intervention on health behaviors and HIV/AIDS treatment management [24]; a study among PLWHA using a novel M-health intervention named the CHAMPS [25]; and a systematic review conducted on mobile health for adherence to antiretroviral therapy among PLWHA [26].

It’s important to note that there is a lack of existing data on medication time adherence from previous related studies, making this research novel and timely. Good adherence to the scheduled drug intake is crucial for the optimal functioning of antiretroviral therapy (ART) and for maintaining the viral load at an undetectable level.

## Conclusion

It is crucial to keep people living with HIV/AIDS (PLWHA) connected to healthcare and ensure they adhere to their medication. This is a top priority for both healthcare and public health, as it can help reduce mortality, co-morbidities, and transmission of the virus. The use of interactive SMS and phone call interventions is a modern and effective approach to improve medication-time adherence. In the test group, m-health interventions have shown to significantly improve the adherence to scheduled medication intake times for PLWHA.

### Recommendations

Mobile health should be integrated with standard care for people living with HIV/AIDS to achieve optimal results. Extensive research on medication adherence is essential as it appears to be lacking.

## Electronic supplementary material

Below is the link to the electronic supplementary material.

Supplementary Material 1

Supplementary Material 2

## Data Availability

No datasets were generated or analysed during the current study.
