# Supplementary material for: Brief communication: Effect of mobile health intervention on medication time adherence among people living with HIV/AIDS receiving care at selected hospitals in Owerri, Imo State Nigeria
Source: AIDS Res Ther. 2024 Oct 24;21:75. doi: 10.1186/s12981-024-00653-0 (PMC11515841; doi:10.1186/s12981-024-00653-0)
Supplement: Supplementary file 1 — Supplementary Material 1 [file 12981_2024_653_MOESM1_ESM.docx]

**ACTG ADHERENCE QUESTIONNAIRE**

|  |  |
| --- | --- |
|  |  |
|  | |

Date __________________ **Self Interviewer Both**

How Administered? 1 2 3

| This page is to be completed by the patient and the researcher together  **Section B (drug dosage and frequency)**    **You are currently taking the following drugs at the frequency and doses listed.**   \| Study Drug Name/Dose \| # Pills Each Time (Pills Each Dose) \| # Times Per Day \| \| --- \| --- \| --- \| \|  \|  \|  \| \|  \|  \|  \| \|  \|  \|  \| \|  \|  \|  \| \|  \|  \|  \| \|  \|  \|  \| \|  \|  \|  \| |
| --- | --- | --- | --- | --- | --- | --- | --- | --- | --- | --- | --- | --- | --- | --- | --- | --- | --- | --- | --- | --- | --- | --- | --- | --- |
|  |
|  |

**INSTRUCTIONS:** Complete this worksheet with the patient**.**

The answers you give on this form will be used to plan ways to help other people who must take pills on a difficult schedule. Please do the best you can to answer all the questions. If you do not wish to answer a question, please draw a line through it. If you do not know how to answer a question, ask the researcher to help. Thank you for helping in this important study.

**PATIENT ONLY continue here**

**Section C (Dosage Adherence)**

The next section of the questionnaire asks about your HIV study medications that you took over the last six days (baseline) / during the four months study period (Post intervention).

Most people with HIV have many pills to take at different times during the day. Many people find it hard to always remember their pills:

• Some people get busy and forget to carry their pills with them.

• Some people find it hard to take their pills according to all the instructions, such as “with meals,” or “on an empty stomach,” “every 8 hours,” “with plenty of fluids.”

• Some people decide to skip doses to avoid side effects or to just not be taking pills that day.

We need to understand how people with HIV are really doing with their pills. Please tell us what you are **actually** doing. Don’t worry about telling us that you don’t take all your pills. We need to know what is really happening, not what you think we “want to hear.

This section of the questionnaire asks about your ability to take your medication at the scheduled time. Please complete the following table by filling in the boxes below.

1. . Most anti-HIV medications need to be taken on a schedule, such as “2 times a day” or “3 times a day” or “every 8 hours.” How closely did you follow your specific schedule over the past six days/ during the study intervention?

Never Some Of About Half Most Of All Of the time

The Time Of The Time The Time The Time

0 1 2 3 4

1. How often were you able to maintain to the two/one hour window for scheduled drug intake agreed on at baseline.

Never Some Of About Half Most Of All Of the time

The Time Of The Time The Time The Time

0 1 2 3 4

1. Some people find that they forget to stick to their scheduled drug intake time over the week-ends. Did you miss any of your anti-HIV medications during the weekends throughout the intervention period— Saturdays or Sundays?

1 Yes 2 No

1. If Yes, how often did you miss it during the weekends?

Some Of About Half Most Of All Of the time

Of The Time The Time The Time

0 1 2 3 4

1. When was the last time you missed taking any of your medications at the correct scheduled time? Check one.

5 Within the past week

4 1-2 weeks ago

3 2-4 weeks ago

2 1-3 months ago

1 More than 3 months ago

0 Never skip medications or not applicable
