# Supplementary material for: Brief communication: Effect of mobile health intervention on medication time adherence among people living with HIV/AIDS receiving care at selected hospitals in Owerri, Imo State Nigeria
Source: AIDS Res Ther. 2024 Oct 24;21:75. doi: 10.1186/s12981-024-00653-0 (PMC11515841; doi:10.1186/s12981-024-00653-0)
Supplement: Supplementary file 2 — Supplementary Material 2 [file 12981_2024_653_MOESM2_ESM.docx]

**CHECKLIST**

**Time adherence**

|  | **a** | **b** | **c** | **Percentage of time adherence** |
| --- | --- | --- | --- | --- |
| **Name /ID No** | **I took my medication at the correct scheduled time for the past six days.** | **I took my medication at the correct scheduled time for just one day, 2 days or 3 days, 4days, 5 days but the complete 6 days.** | **I was unable to take my medication at the correct scheduled time in the past 6 days.** | **Percentage of time adherence** |
|  |  |  |  |  |
|  |  |  |  |  |
|  |  |  |  |  |
|  |  |  |  |  |
|  |  |  |  |  |
|  |  |  |  |  |
|  |  |  |  |  |
|  |  |  |  |  |
|  |  |  |  |  |
|  |  |  |  |  |
|  |  |  |  |  |
|  |  |  |  |  |
